# Synthesis and Weak Hydrogelling Properties of a Salt Resistance Copolymer Based on Fumaric Acid Sludge and Its Application in Oil Well Drilling Fluids

**DOI:** 10.3390/gels8050251

**Published:** 2022-04-20

**Authors:** Zhongjin Wei, Fengshan Zhou, Sinan Chen, Wenjun Long

**Affiliations:** Beijing Key Laboratory of Materials Utilization of Nonmetallic Minerals and Solid Wastes, National Laboratory of Mineral Materials, School of Materials Science and Technology, China University of Geosciences (Beijing), Beijing 100083, China; 3003190032@cugb.edu.cn (Z.W.); 2103190085@cugb.edu.cn (S.C.); longwj@cugb.edu.cn (W.L.)

**Keywords:** fumaric acid sludge (FAS), free radical copolymerization, filtrate loss reducer, organic wastewater of phthalic anhydride, oil well drilling fluids

## Abstract

Fumaric acid sludge (FAS) by-produced from phthalic anhydride production wastewater treatment contains a large amount of refractory organic compounds with a complex composition, which will cause environmental pollution unless it is treated in a deep, harmless manner. FAS included saturated carboxylic acid, more than 60%, and unsaturated carboxylic acid, close to 30%, which accounted for the total mass of dry sludge. A new oil well drilling fluid filtrate loss reducer, poly(AM-AMPS-FAS) (PAAF), was synthesized by copolymerizing FAS with acrylamide (AM) and 2-acrylamide-2-methyl propane sulfonic acid (AMPS). Without a refining requirement for FAS, it can be used as a polymerizable free radical monomer for the synthesis of PAAF after a simple drying process. The copolymer PAAF synthesis process was studied, and the optimal monomer mass ratio was determined to be AM:AMPS:FAS = 1:1:1. The temperature resistance of the synthesized PAAF was significantly improved when 5% sodium silicate was added as a cross-linking agent. The structural characterization and evaluation of temperature and complex saline resistance performance of PAAF were carried out. The FT-IR results show that the structure of PAAF contained amide groups and sulfonic acid groups. The TGA results show that PAAF has good temperature resistance. As an oilfield filtrate loss reducer, the cost-effective copolymer PAAF not only has excellent temperature and complex saline resistance, the API filtration loss (FL) was only 13.2 mL/30 min after 16 h of hot rolling and aging at 150 °C in the complex saline-based mud, which is smaller compared with other filtrate loss reducer copolymers, but it also has little effect on the rheological properties of drilling fluid.

## 1. Introduction

Phthalic anhydride is usually produced by the naphthalene method [1], which is produced by the synthesis of naphthalene and o-xylene through the process of oxidation, isomerization, drying and crystallization, etc. The organic tail gas produced in the production of phthalic anhydride becomes acidic organic wastewater after absorbing washing water, and then the catalyst thiourea was added and the maleic acid in the wastewater was transformed into fumaric acid. After cooling, crystallization, centrifugation, and other operations, the fumaric acid production wastewater is produced.

**Figure 1:**
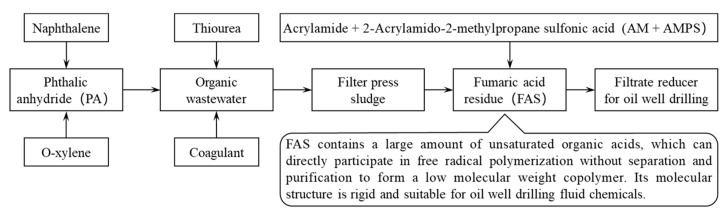
Flowchart of the source of FAS and its high-value utilization method.

A large amount of fumaric acid production wastewater is produced during the production of phthalic anhydride, and the COD of these organic wastewater is about 6000–12,000 mg/L [2], which requires the addition of flocculants for flocculation and sedimentation, and the flocculation products are pressed and filtered to obtain press-filtered sludge. Fumaric acid sludge (FAS) can be obtained by drying the press-filtered sludge, as shown in Figure 1. A plant producing about 150,000 tons of phthalic anhydride will produce about 4000 tons of FAS every year, and this FAS sludge may contain hazardous chemicals such as naphthoquinone, naphthalene, maleic anhydride, etc., which will cause environmental pollution unless it is treated in a deep, harmless manner. Therefore, utilized FAS as a high-value resource has important environmental and economic significance. 

The specific composition of FAS is different due to different synthetic process routes, operation process parameters of different manufacturers, organic wastewater treatment process, etc. The substances contained in FAS generally include phthalic acid, benzoic acid, citric acid, maleic acid, fumaric acid and other organic acids, mainly including fumaric acid and maleic acid.

Due to the high value of fumaric acid, many researchers try to separate and purify fumaric acid from FAS [3,4,5]. However, it is difficult to separate high-purity fumaric acid from FAS, and the process is very complicated, going through the steps of washing and sedimentation, decolorization, filtration, transposition reaction, drying, filtrate treatment, etc. These processing methods require many instruments, high energy consumption, high treatment cost, and low economic efficiency; finding a cost-effective way to treat FAS has become a challenge.

Some researchers have studied the use of FAS to produce polyol unsaturated polyester resin to produce artificial marble/granite and foamed resin for home decoration building materials, but failed to realize industrial application [6]. In addition, only unsaturated carboxylic acids (about one-third) in FAS are utilized in the production of unsaturated polyester resin. Even if the average price of maleic acid and fumaric acid industrial products is 6000 CNY/t, the utilization value is only about 2000 CNY/t. How to realize the utilization of saturated carboxylic acid (about three-fifths) in FAS is more economical. 

Unsaturated carboxylic acids account for about one-third of FAS, while saturated carboxylic acids account for two-thirds. This compositional feature determines that the utilization of FAS cannot only consider unsaturated dicarboxylic acids, and the value of saturated carboxylic acids is equally important, or even more important. Citric acid and its aluminum salts are widely used in oilfield water injection scale inhibitors and cross-linking agents for polymer hydrogels [7,8,9,10]. Therefore, the direct utilization of FAS may be the best in the field of oilfield chemicals.

Drilling fluid, known as the “blood” in drilling engineering, has the functions to carry cuttings, protect the well-bore wall, balance formation pressure and reduce drilling tool wear [11]. With the continuous low price of oil, the direction of development of drilling fluid additives highlights the utilized resources of wastes to reduce the production cost of the additives [12,13,14]. If FAS can be directly utilized to prepare drilling fluid additives, it is not only an important process method to solve the clean production of phthalic anhydride, but also an effective way to reduce the production cost of drilling fluid additives. 

Oilfield filtrate loss reducer is the core additive of drilling fluid, which can form a thin and dense mud cake at the well wall, reduce the amount of drilling fluid filtrate loss penetration into the formation interior, avoid well collapse, and ensure safe oilfield production. Filtration control is an important property of a drilling fluid, particularly when drilling through permeable formations, where the hydrostatic pressure exceeds the formation pressure. Commonly used filtrate loss reducer includes cellulose derivatives, starch derivatives, humic acids, synthetic resins, and sulfonic acid-based multipolymer [15,16]. Both fumaric acid and maleic acid contain –C=C– in their molecular structures, and it is feasible to utilize FAS as a polymeric monomer for the preparation of multiple copolymers and then as a filtrate loss reducer for oilfield drilling applications. Many researchers have utilized fumaric acid and maleic acid to synthesize oilfield chemical additives in recent years [17,18,19].

FAS contains a large amount of fumaric anhydride and maleic anhydride; their molecular structure can easily form a rigid ring structure through hydrogen bonding, which results in low polymerization activity (low reactivity rate) and difficulty in forming a large molecular weight in a free radical polymerization reaction (such as hydrolyzed poly-maleic anhydride (HPMA), a polymer-scale inhibitor with excellent performance, the short molecular chain of which is due to the carboxyl group formed by hydrolysis, which makes it easier to form carboxyl salts with metal ions). Therefore, polymer containing FAS has the function of small-molecule viscosity reducer. On the other hand, the ring structure in the molecular structure greatly increases the molecular rigidity of the polymer, which makes the polymer relatively superior in salt resistance and high-temperature resistance to a certain extent. The industrial by-product FAS used in the preparation of polymer filtrate loss reducer can not only reduce the cost of the polymer, but also improve the temperature and salt resistance of filtrate loss reducer. Therefore, FAS used in the synthesis of polymer filtrate loss reducer has a high application value.

In order to realize the efficient utilization of industrial byproduct FAS and prepare a low-cost filtrate loss reducer with temperature and salt resistance, in our work, firstly, a series of qualitative and quantitative analyses of FAS were carried out; and then put forward a method of industrialized utilization of FAS as a high-value resource; then, a temperature and complex saline resistance filtrate loss reducer based on FAS was successfully synthesized; finally, the performance evaluation and structural characterization of the synthesized copolymer were carried out (Figure 1).

## 2. Results and Discussion

### 2.1. Component Analyses of FAS by Modern Instrumental Analysis Method

**Figure 2:**
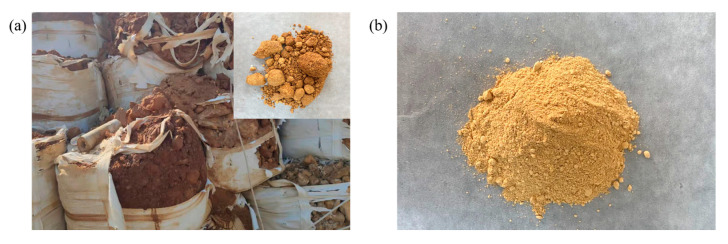
Appearance comparison of FAS: (**a**) original appearance; (**b**) after drying and crushing.

FAS appears as a dark yellow massive solid, which can easily absorb water and agglomerate when exposed to air, turning to light yellow fine powder after drying and crushing, as shown in Figure 2. In order to find out the resource utilization method of FAS, a series of qualitative and quantitative analyses of FAS were carried out: FT-IR is used to detect possible functional groups; ^1^H-NMR is used to calculate the degree of unsaturation of different substances in FAS; XRD is used to analyze the main phases of FAS; XRF is used to analyze the elements contained in FAS and the content of each element; GC-MS and Py-GC-MS are used to determine possible volatiles and pyrolysis products in FAS; and LC-MS is used to detect the content of water-soluble substances in FAS. Then, the detailed components and ratios of FAS were obtained, as shown in Table 1.

It can be seen from Table 1 that the content of unsaturated carboxylic acid is 33.69%, the content of saturated carboxylic acid is 64.21%, and the content of diprotic carboxylic acid is 51.04%. For oilfield chemicals, the saturated acids in FAS can be applied to oilfield water injection scale inhibitor and cross-linking agent for polymer hydrogels, and unsaturated acids can be used to synthesize filtrate reducer. The application of FAS in the field of oilfield chemicals can effectively improve the utilization rate of FAS. 

AM (acrylamide) and AMPS (2-acrylamide-2-methyl propane sulfonic acid) are two commonly used monomers for the synthesis of polyacrylamide, since their molecular structures contain functional groups such as amide and sulfonic acid groups, which are often introduced into the molecular structure of oilfield filtrate loss reducer to improve their temperature and saline resistance [20,21]. In recent years, many researchers have used AM/AMPS in copolymerization to synthesize copolymers and applied them in various fields [22,23,24,25]. 

Therefore, we propose an industrialized utilization of FAS: due to the presence of unsaturated carboxylic acids, their molecular structure contains –C=C–, and the FAS can be utilized as a polymerizable free radical monomer and copolymerized with AM/AMPS to synthesize copolymer as oilfield drilling filtrate loss reducer. In this way, the role of saturated carboxylic acid and unsaturated carboxylic acid in FAS can be fully exerted, and the utilization rate of FAS can reach 97.9%.

### 2.2. Structural Characterization of PAAF

#### 2.2.1. FT-IR

**Figure 3:**
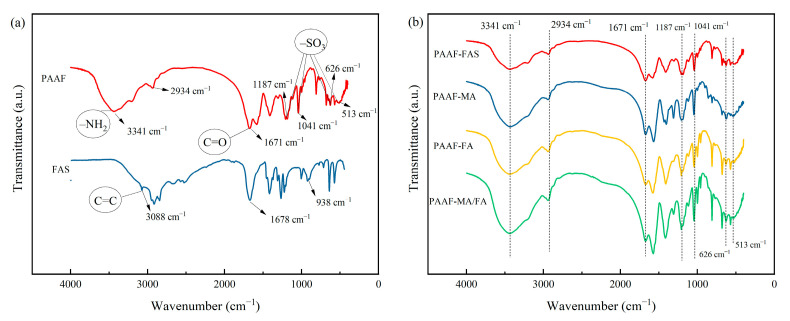
Infrared spectrum comparison: (**a**) PAAF and FAS; (**b**) PAAF-FAS\PAAF-MA\PAAF-FA\PAAF-MA-FA.

The infrared spectrum of PAAF and FAS are shown in Figure 3a. In the FT-IR spectrum of FAS, the absorption peak at 1678 cm^−1^ corresponds to the characteristic absorption peak of C=O, and 3088 and 934 cm^−1^ correspond to the stretching vibration in =CH and the –OH non-planar wobble vibration, respectively [26]. While in the FT-IR spectrum of PAAF, the absorption peak at 3088 cm^−1^ becomes very flat, which indicates that the stretching vibration of =CH is very weak in the PAAF structure and the C=C in the raw material AM/AMPS/FAS structure has been broken. In addition, in the FT-IR spectrum of PAAF, the absorption peaks at 3341 and 1671 cm^−1^ correspond to the N–H bond and C=O bond stretching vibration peaks, respectively [27]; 2934 cm^−1^ corresponds to the stretching vibration of saturated –CH [27]; the peaks appearing at 1187, 1041, 626, and 513 cm^−1^ are the –SO_3_ absorption characteristic peak [28]. The characterization results show that the polymer PAAF structure contains groups such as –CONH_2_, –SO_3_, etc. These functional groups are introduced by copolymerization through C=C breakage in the raw material AM/AMPS/FAS structure.

Figure 3b gives the FT-IR spectrum of PAAF prepared with the following different monomers: FAS and MA (analytically pure), FA (analytically pure), and MA/FA (analytically pure). It can be seen from the figure that the FT-IR spectrum of this series of PAAF does not differ much, and the positions of the corresponding absorption characteristic peaks of the main functional groups are basically the same. It means that PAAF synthesized with unpurified FAS has the same functional groups as that synthesized with analytically pure compounds, and FAS does not need to be refined and can be utilized directly as a polymeric monomer to copolymerize with other monomers.

#### 2.2.2. TGA

**Figure 4:**
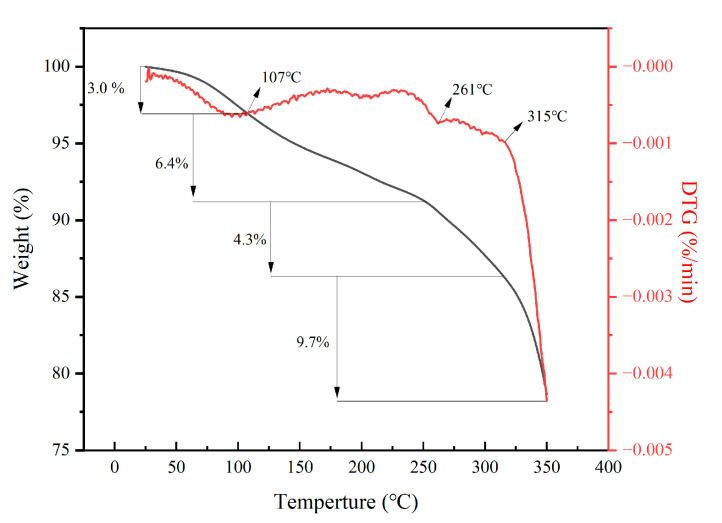
TGA-DTGA curve of PAAF.

Figure 4 shows the TGA-DTGA curve of PAAF. As can be seen from the figure, the decomposition of PAAF is divided into four stages. The first stage occurs at room temperature to 107 °C, which is the free water evaporation stage. The evaporation of intramolecular and intermolecular water corresponds to a weight loss of 3.0%. The second stage occurs at 107 to 261 °C; at this stage, the amide group undergoes thermal acyl-amination reaction [29], resulting in a weight loss of 6.4%. The third stage occurs at 261 to 315 °C, the –C–C– bond on the side chain of PAAF begins to break, and the molecular structure on the side chain begins to detach from the main chain of PAAF and undergo carbonization, corresponding to a weight loss of 4.3%. The fourth stage occurs at 315 to 350 °C; in this stage, the –C–C– bond in the main chain of the PAAF molecular structure begins to break, and the macromolecular chain of PAAF begins to break into small molecular chains, corresponding to a weight loss of 9.7%. From the TGA-DTGA results, it can be seen that the molecular structure of PAAF began to decompose from 261 °C, and the general oil well filtrate loss reducer use environment is lower than 200 °C, so as an oil well filtrate loss reducer, PAAF can have a good temperature resistance.

### 2.3. Performance Evaluation of PAAF

#### 2.3.1. Effect of Monomer Mass Ratio

To improve the utilization rate of FAS, a series of PAAF were synthesized by gradually increasing the proportion of FAS in the total monomer (0%, 20%, 25%, 33%, 50%) while keeping the mass ratio AM:AMPS = 1:1 and the total mass of the three monomers AM/AMPS/FAS constant, which were recorded as PAAF110, PAAF221, PAAF332, PAAF111, and PAAF112. Weigh two portions of a certain mass of PAAF and add them to two groups of complex saline-based mud, respectively. One portion was stirred at high speed for 20 min, and then stirred at high speed for 10 min after 24 h of maintenance to determine the room temperature filtration loss FL_API_ and rheological parameters; the other group was stirred at high speed for 20 min, loaded into a high-temperature aging tank, and hot rolled and aged at 150 °C for 16 h. After it cools, take it out and stir at high speed for 10 min, and then determine the room temperature filtration loss FL_API_ and rheological parameters.

**Figure 5:**
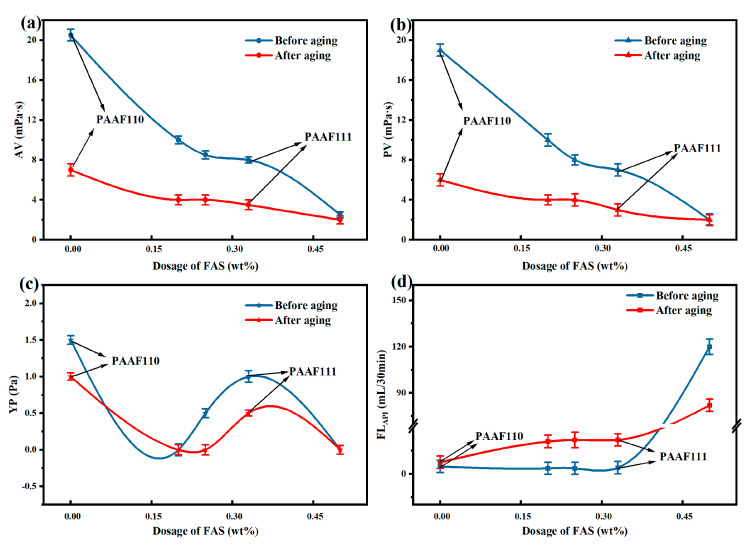
Effect of monomer ratio on the performance of PAAF: (**a**) effect of monomer ratio on AV; (**b**) effect of monomer ratio on PV; (**c**) effect of monomer ratio on YP; (**d**) effect of monomer ratio on FL_API_.

The AV, PV, YP, and FL_API_ of the complex saline-based mud were all higher after 24 h of room temperature maintenance than after aging at 150 °C for 16 h (Figure 5), indicating that part of the carbon chain structure of PAAF was destroyed at 150 °C, leading to a decrease in its rheological properties and filtration loss reduction performance. With the increase in FAS proportion, the AV and PV of the complex saline-based mud showed a decreasing trend (Figure 5a,b), but FL_API_ did not increase significantly (Figure 5d), indicating that the addition of a certain proportion of FAS can significantly reduce the viscosity of the complex saline-based mud, and the filtrate loss reduction performance will not be significantly reduced. However, there is a significant increase in FL_API_ as the proportion of FAS increases to 50% (Figure 5d), comparing PAAF111/PAAF112, from 22.0 to 82.0 mL after hot rolling at 150 °C, which can no longer meet the technical requirements of oilfield filtrate loss reducer (≤ 25 mL). It is possible that the proportion of FAS in the total amount of monomers is too high, and FAS contains a large amount of fumaric acid, and the double carboxyl group of fumaric acid is located on the opposite side of the carbon-carbon double bond, which has a large polymerization site resistance and is not conducive to the polymerization between the monomers, making the degree of polymerization between AM/AMPS/FAS lower, resulting in a sharp decrease in the performance of the synthesized PAAF to reduce filtration loss. Therefore, considering the utilization rate of FAS and filtration loss reduction performance, the synthesized PAAF can meet the requirements of oilfield filtrate loss reducer and maximize the utilization of FAS when the mass ratio AM:AMPS:FAS = 1:1:1.

#### 2.3.2. Effect of Cross-Linking Agent

Unsaturated carboxylic acids account for about one-third of FAS, while saturated carboxylic acids account for about three-fifths. This compositional feature determines that the utilization of FAS cannot only consider unsaturated dicarboxylic acids, and the value of saturated carboxylic acids is equally important, or even more important. Citric acid and its aluminum salts are widely used in oilfield water injection scale inhibitors and cross-linking agents for polymer hydrogels, easy-to-form weak hydrogels. In the reaction of synthesizing copolymers, the saturated polyacids in FAS may be thermally cross-linked with small-molecule polymers to form weak gels with a blocking effect. However, due to the cross-linking formed by covalent bonds, its cross-linking strength is relatively weak. Although it has an effect on improving the fluid loss reduction performance at room temperature, it is relatively weak in improving the high-temperature resistance of the copolymer. We conducted an experimental exploration to improve the high temperature resistance of FAS-based copolymers by using silicates as cross-linking agent.

Sodium silicate readily forms polysilicate at a certain pH range and can be applied in polymerization reactions to strengthen the structure of polymer molecules [30,31,32,33,34]. Under the condition of monomer mass ratio AM:AMPS:FAS = 1:1:1, different masses of sodium silicate were added to the solution as cross-linking agent to improve the temperature resistance of PAAF, and the masses of sodium silicate were 0.0, 1.5, 3.0, 6.0, and 12.0 g. After adding different masses of sodium silicate to synthesize the series of PAAF, the FL_API_ and rheological parameters of the complex saline-based mud were measured based on the previously introduced test method for temperature and salt resistance, and the test results are shown in Table 2.

With the addition of cross-linking agent, the FL_API_ before aging does increase, but it is obvious that the FL_API_ after aging first decreases significantly, and then starts to increase when the addition of cross-linking agent exceeds 6.0 g, which means that the cross-linking agent can improve PAAF’s ability to reduce filtration in high temperature, but adding too much cross-linking agent will greatly reduce the hydration ability of PAAF molecules, resulting in a decrease in its ability to reduce filtration. Therefore, there is an optimal mass for the cross-linking agent. Combining the data of FL_API_ before aging and FL_API_ after aging, we believe that PAAF’s ability to reduce filtration is the best when the addition of the cross-linking agent is 3 g. On the other hand, the YP value changes significantly with the increase in the crosslinking agent. This is because the PAAF molecules form a certain network structure through the crosslinking reaction, which greatly enhances the yield value. It also happens that when the addition of cross-linking agent is about 3.0 g, the YP before aging and after aging is relatively high, which is good for the cuttings carrying ability of the drilling fluid. Therefore, it is very necessary to optimize the amount of cross-linking agent in general, and it has indeed achieved better results.

As can be seen from Table 2, the rheological parameters AV, PV and YP of the complex saline-based mud did not change much after the addition of a certain mass of sodium silicate, indicating that the addition of sodium silicate hardly affected the rheological properties of PAAF. There was a significant decrease in FL_API_ after hot rolling and aging at 150 °C for 16 h, FL_API_ decreased from 22.0 to 16.0 mL/30 min after adding 1.5 g of sodium silicate, and FL_API_ was lowest at 13.2 mL/30 min when 3.0 g sodium silicate was added, indicating that a certain mass of sodium silicate could improve the temperature resistance of PAAF. However, with the further increase in sodium silicate mass (increased to 6.0 g), the FL_API_ after hot rolling and aging increased sharply instead (from 13.2 mL/30 min to 66.0 mL/30 min), indicating that the addition of too much sodium silicate hinders the polymerization between AM/AMPS/FAS. It makes the polymerization of PAAF decrease, which leads to the carbon chain of PAAF to break easily at high temperatures, thus losing the function of reducing filtration loss.

Therefore, the addition of 3.0 g sodium silicate (5% of the total monomer mass) can make the monomers on the main chain of PAAF cross-link with each other and ensure that the structure of PAAF is not easily destroyed at high temperature; thus, the temperature resistance of PAAF can be improved.

#### 2.3.3. Effect of FAS on Rheological Properties

**Figure 6:**
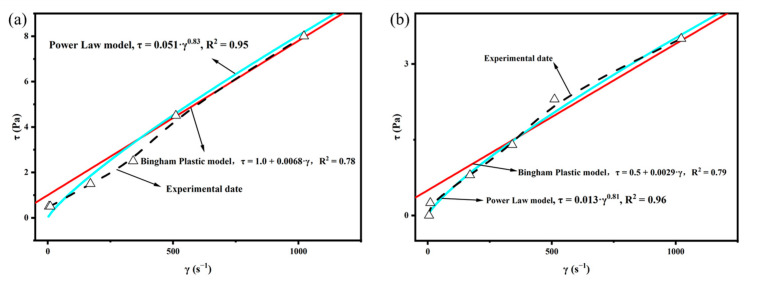
Rheological model cure: (**a**) rheological models applied to viscometric data obtained before aging; (**b**) rheological models applied to viscometric data obtained after aging.

In order to more scientifically evaluate the rheological properties of the complex saline-based mud used in this study, the Bingham Plasticity Model and Power Law Model were applied to the viscometric data obtained before and after aging. As can be seen from Figure 6, whether it is before or after aging, the Power Law Model is more consistent with the experimental data curve than the Bingham Plastic Model. Therefore, we consider the complex saline-based mud to be fitted with the Power Law Model.

According to Power Law Model: τ= Kγ^n^, where τ is the shear stress, γ is the shear rate, n is the fluidity index, and K is the consistency coefficient. The value of n reflects the shear dilution performance of drilling fluid, and K is related to the viscosity and sheared force of drilling fluid. The K value reflects the pumpability of drilling fluid; the large value of K will make it difficult to repump. In order to study the effect of FAS on the rheological properties of drilling fluid, we added two additives to the complex saline-based mud, PAAF110 (without FAS) and PAAF111 (mass ratio AM:AMPS:FAS = 1:1:1), and tested the changes of rheological parameters of composite brine base mud under different additives, as shown in Table 3.

It can be seen from Table 3 that at 25 °C, the AV and PV values of based mud with PAAF110 are much greater than those of based mud with PAAF111, which indicates that the based mud with PAAF111 has better fluidity; at 150 °C, the RYP value and n value of based mud with PAAF110 are consistent with based mud with PAAF111, indicating that the two additives have the same shear ability, and there is no difference in the ability to carry cuttings. The K value of based mud with PAAF110 is half of that of based mud with PAAF111, which means that based mud with PAAF110 is more viscous, has worse pumpability, and is more difficult to pump the drilling fluid to the ground. Therefore, the PAAF111 synthesized based on FAS can not only maintain the ability of drilling fluid to carry cuttings, but also make the drilling fluid have better fluidity and facilitate pumping.

In general, polymer filtrate loss reducer has a positive effect on rheological properties, the value of n is appropriate to the control at 0.4–0.7, and the value RYP is appropriate to the control at 0.36–0.48 Pa/mPa·s. Obviously, the effect of PAAF and general polymer filtrate loss reducer on rheological properties is not the same, because PAAF is a dilution-type filtrate loss reducer with a relatively smaller molecular weight. This can be clearly verified from the results of previous studies on China’s most famous polymer diluent, XY-27 (ultra-low-molecular-weight poly(AM-AA)) [35].

After PAAF111 is added, the value of RYP and K is relatively small. On the one hand, the dilution of PAAF weakens the network structure formed between polymer and bentonite particles, which reduces the structural viscosity (intrinsic viscosity) of drilling fluid; On the other hand, a certain amount of saturated organic acids (citric acid, phthalic acid, etc., which will not participate in the polymerization of PAAF) contained in FAS will form small molecular organic salts with metal ions on the end face of bentonite particles under high-temperature conditions, compress the electric double layer on the surface of bentonite particles, and appear with the tendency of high temperature passivation of bentonite particles, which affects the stability of the bentonite colloidal system and leads to poor thixotropy.

However, this paper only studies the effect of a single PAAF in the bentonite-based mud on the rheological properties, in order to understand the influence of the difference of PAAF itself on the rheological properties of the drilling fluid. In practical industrial applications, drilling fluid is composed of many kinds of functional additives (sometimes even more than ten kinds of additives), and the effect of different additives on drilling fluid rheological properties will be affected by their interaction Therefore, the rheological properties of the drilling fluid system is the result of the joint action of various additives, and it is also the result of the coupling effect of various additives complementing each other. It is not difficult to understand that even if the effect of a single additive on the rheological properties is negative, the combined effect in the actual drilling fluid may still be positive. Examples abound in this regard, such as Fe-Cr-lignosulfonate (FCLS), the most effective diluent in brine drilling fluid systems, which has exactly the same effect on the rheological properties of drilling fluid systems [35].

#### 2.3.4. Comparison of By-Product FAS with Analytical Pure MA and FA

**Figure 7:**
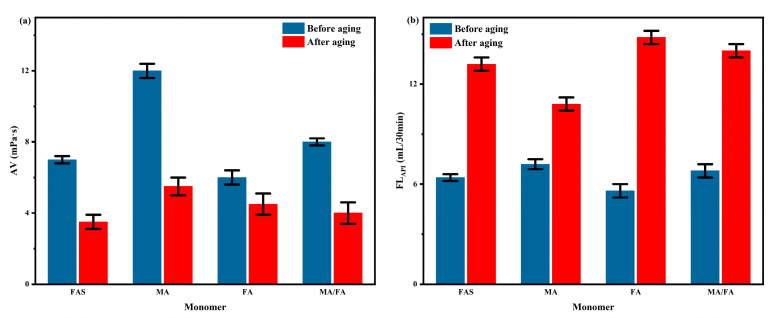
Effect of different monomers on the performance of PAAF: (**a**) effect of different monomers on AV; (**b**) effect of different monomers on FL_API_.

Under optimal experimental conditions, FAS, maleic acid (MA, analytical pure), fumaric acid (FA, analytical pure), and MA:FA = 1:3 were used to co-polymerize with AM/AMPS to form a series of PAAF, respectively. The AV and FL_API_ of the complex saline-based mud after adding the series PAAF were tested before and after hot rolling and aging at 150 °C for 16 h according to the performance evaluation method, and the test results are shown in Figure 7.

From the AV values before hot rolling and aging (Figure 7a), the apparent viscosity of MA is the highest (12.0 mPa·s) and the apparent viscosity of FA is the lowest (6.0 mPa·s), which indicates that MA has the highest degree of polymerization with AM/AMPS and the highest molecular weight of the synthesized PAAF compared to FAS and FA. From the FL_API_ after hot rolling and aging (Figure 7b), the PAAF synthesized using MA has the best reduce filtration loss performance (10.8 mL, the double carboxyl group of MA is located on the same side of the carbon-carbon double bond, and the site resistance of polymerization is smaller than FA, which is easier to polymerize). There was little difference in the filtration loss reduction effect between PAAF synthesized directly using FAS and PAAF synthesized at MA:FA = 1:3 (13.2 mL and 14.0 mL, respectively), which indicates that the components of FAS other than fumaric acid and maleic acid do not affect the polymerization between FAS and AM/AMPS. Therefore, there is no complicated refining requirement, and FAS can be used directly as polymerizable free radical monomer for the synthesis of oilfield filtrate loss reducer PAAF.

#### 2.3.5. Filtration Properties of PAAF

**Figure 8:**
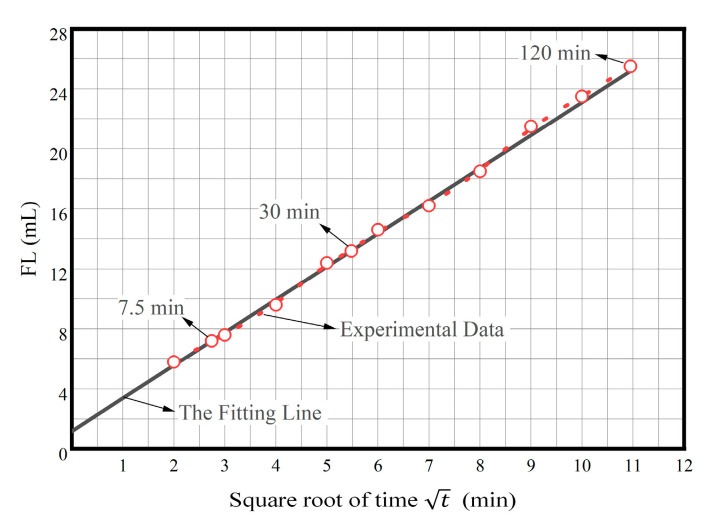
The plot of relationship between square root of time and FL (after aging at 150 °C).

According to Darcy’s law [35]: dV_f_/dt = KA△p/(μh_mc_), where dV_f_/dt is filtration loss rate, cm^3^/s; K is mud cake permeability, μm^2^; A is percolation area, cm^2^; △p is percolation pressure, 105 Pa; μ is filtration loss viscosity, 0.1 mPa·s, h_mc_ is mud cake thickness, cm; V_f_ is filtration loss (FL), cm^3^; t is percolation time, s. We can infer that FL and $\sqrt{t}$ should be proportional. Figure 8 shows the plot of the relationship between square root of time and FL (After aging at 150 °C). As $\sqrt{t}$ increases, the change trend of FL is almost linear. We take the FL at 7.5 and 30 min, respectively, and fit a straight line; we can find that the values of FL are almost all on this straight line. Therefore, it can be considered that FL is proportional to $\sqrt{t}$, and the filtration loss characteristics of PAAF follow Darcy’s law. On the other hand, we can find that this straight line is not at the origin and has a positive intercept, indicating that before the mud cake is formed, there is a sudden spurt of initial filtration loss, that is, spurt loss. When spurt loss occurs, the spurt loss molecules can quickly enter the bottom of the thin cuttings formed by the broken rock of the drill bit, which can help to strip the thin cuttings from the rock and wash away immediately, or make them not closed, which is beneficial to increase the mechanical rotational speed.

The ability of the mud to seal permeable formations exposed by the bit with a thin, low-permeability filter cake is another major requirement for successful completion of the hole. Because the pressure of the mud column must be greater than the formation pore pressure in order to prevent the inflow of formation fluids, the mud would continuously invade permeable formations if a filter cake were not formed.

The rate of filtration and the increase in cake thickness depend on whether or not the surface of the cake is being subjected to fluid or mechanical erosion during the filtration process. When the mud is static, the filtrate volume and the cake thickness increase in proportion to the square root of time (hence, at a decreasing rate). Under dynamic conditions, the surface of the cake is subjected to erosion at a constant rate, and when the rate of growth of the filter cake becomes equal to the rate of erosion, the thickness of the cake and the rate of filtration remain constant. In the well, because of erosion by the mud and because of mechanical wear by the drill string, filtration is dynamic while drilling is proceeding; however, it is static during round trips. All routine testing of filtration properties is made under static conditions because dynamic filtration tests and static tests under high temperature and high pressure (HTHP) are time-consuming and require elaborate equipment. Thus, filtration rates and cake thicknesses measured in surface tests correlate only approximately to those prevailing down-hole and can be grossly misleading. The permeability of the filter cake, which may readily be calculated from static test data, is a better criterion because it is the fundamental factor controlling both static and dynamic filtration.

#### 2.3.6. Comparison of Filtration-Loss-Controlling Ability of Popular Copolymers

We selected several popular low-molecular-weight copolymer filtrate loss reducers (Na-PAN, NH_4_-PAN and PAMAP) and compared their filtration loss in different based mud. The Na-PAN is hydrolyzed polyacrylonitrile sodium salt, which is a commonly used industrial filtrate loss reducer. It is insensitive to NaCl and has good filtration loss performance for based mud containing NaCl. The NH_4_-PAN is also an industrial filtrate loss reducer, owing to the fact that NH_4_^+^ released by NH_4_-PAN in the drilling fluid can be embedded in the shale; it can not only reduce the filtration loss, but also have a certain effect of preventing well collapse. The PAMPAP is a copolymer obtained by copolymerization of AM/AMPS/AA/AP and has a similar molecular structure to PAAF [27]. The FL data for these filtrate loss reducers are listed in Table 4.

As can be seen from Table 4, compared with Na-PAN, PAAF has worse filtration loss performance in saturated brine-based mud, while PAAF has better filtration performance in complex saline-based mud, which means that although the PAAF has a weaker NaCl resistance performance, its complex saline resistance performance is much greater than Na-PAN. Compared to NH_4_-PAN, it is clear that PAAF exhibits stronger filtration loss performance both in saturated brine-based mud and in complex saline-based mud. Compared with PAMAP, which is similar in molecular structure, the filtration loss performance of PAAF at room temperature medium pressure is better, but the filtration loss performance of PAAF is not good enough at high temperature high pressure (HTHP), the reason being that PAAF is a small molecule filtrate loss reducer with a dilution function, meaning the molecular weight of PAAF is too small to block the filtration loss channel. Therefore, even a salt resistance filtrate loss reducer such as PAAF, in the complex saline-based mud used in this paper, does not have a good enough HTHP filtration loss performance (FL_HTHP_ is 126 mL).

The danger of relying on the API filter loss as a criterion for downhole dynamic filtration rates is obvious. A treating agent that was recommended on the basis of API test results might give higher rates downhole than another agent that gave a higher API filtrate loss. Worse still, an agent that reduced the API loss might increase the downhole filtration rate.

The HTHP static loss correlated quite well with the short term (30 min) dynamic loss, but had virtually no relationship with the long term (equilibrium) dynamic loss.

Despite its shortcomings, the API static test is the only practical test for control of filtration at the wellsite. However, results should be interpreted in the light of correlations made in the laboratory between API filter loss and dynamic filtration rate.

#### 2.3.7. Mud Cake Properties of PAAF

**Figure 9:**
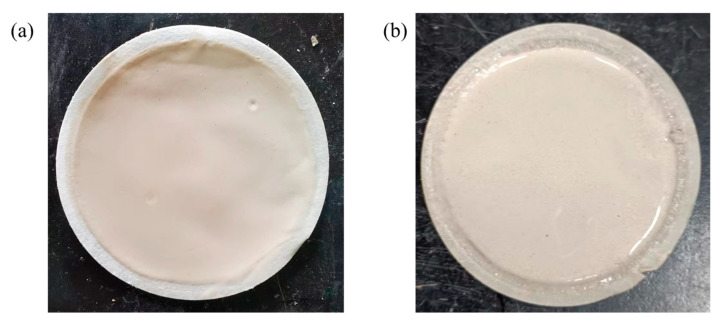
Mud cake produced under different conditions: (**a**) room temperature medium pressure; (**b**) high temperature high pressure (HTHP).

Figure 9 shows the mud cake produced at room temperature medium pressure and the mud cake produced at high temperature high pressure (HTHP). It can be seen that the mud cake produced at room temperature medium pressure is very thin and dense, and is tightly attached to the filter paper, showing a strong toughness, and the thickness of the mud cake is less than 0.5 mm. The mud cake formed at high temperature high pressure is not dense enough, and the thickness of the mud cake is about 1.8 mm. The particles on the surface of the mud cake are evenly distributed, and there is no large particle agglomeration. This shows that PAAF has played a diluting and dispersing role even in high temperature and high pressure (HTHP) condition, so that the bentonite particles do not agglomerate together.

For a mud cake to form, it is essential that the mud contain some particles of a size only slightly smaller than that of the pore openings of the formation. These particles, which are known as bridging particles, are trapped in the surface pores, while the finer particles are, at first, carried deeper into the formation. The bridged zone in the surface pores begins to trap successively smaller particles, and, in a few seconds, only liquid invades the formation. The suspension of fine particles that enters the formation while the cake is being established is known as the “mud spurt”. The liquid that enters subsequently is known as the filtrate.

The permeability of the mud cake depends on the particle size distribution in the mud and on the electrochemical conditions. In general, the more particles there are in the colloidal size range, the lower the cake permeability. The presence of soluble salts in clay muds increases the permeability of the filter cake sharply, but certain organic colloids enable low cake permeabilities to be obtained even in saturated salt solutions. Thinners usually decrease cake permeabilities because they disperse clay aggregates to smaller particles.

#### 2.3.8. Salt Resistance of PAAF

**Figure 10:**
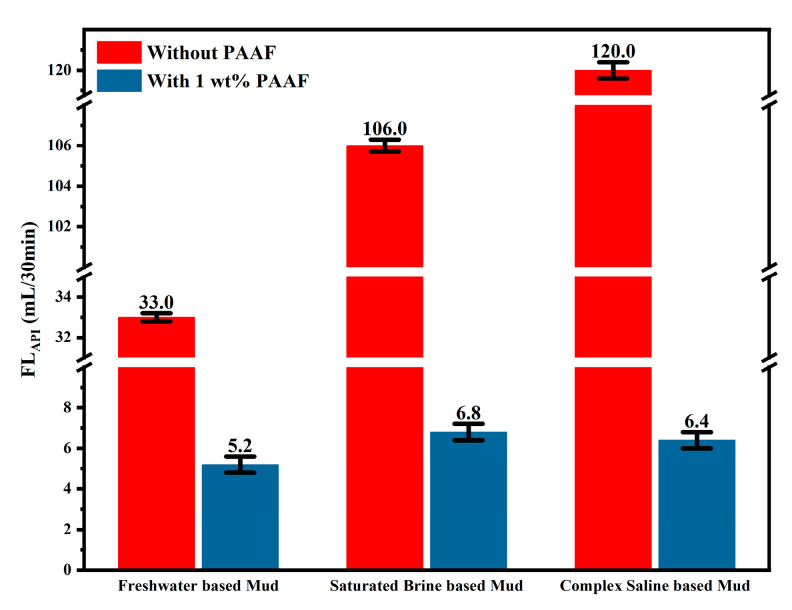
FL_API_ comparison under different based mud (before aging).

Figure 10 shows the comparison of the FL_API_ (before aging) in different based mud. After adding 1wt% PAAF to the based mud, the filtration loss of freshwater-based mud was reduced by 27.8 mL/30 min; the filtration loss of saturated brine-based mud was reduced by 99.2 mL/30 min, and the filtration loss of complex saline-based mud was even lower, 113.6 mL/30 min. This result shows that PAAF exhibits excellent salt resistance. The salt resistance mechanism of PAAF can be explained as follows: On the one hand, the molecular structure of PAAF contains sulfonic acid groups, which are not sensitive to alkaline earth metal ions, so PAAF can also play a good hydration effect in a high-salinity environment, forming a thicker hydration shell, slowing down the loss of water in the mud. On the other hand, a large number of amide groups are distributed in the molecular structure of PAAF, and the amide group has a strong adsorption effect [37], which can eliminate the expansion of the interlayer spacing of bentonite by metal ions, promoting the formation of dense mud cake.

## 3. Conclusions

The FAS is a byproduct of the processing of phthalic anhydride organic wastewater, which is rich in saturated carboxylic acid and unsaturated carboxylic acid. FAS as a free radical monomer can directly participate copolymerization with acrylamide (AM) and 2-acrylamido-2-methylpropane sulfonic acid (AMPS) without separation and purification to form a low-molecular-weight copolymer. Its molecular structure is rigid and suitable for oil well drilling fluid chemicals such as filtrate loss reducer with a very low cost and good comprehensive performance. We achieved the efficient utilization of FAS, and the utilization rate of FAS can reach 97.9%.

In the mass ratio AM:AMPS:FAS = 1:1:1, with the addition of 5 wt% sodium silicate, the prepared copolymer PAAF has the best comprehensive performance. As a filtrate loss reducer for oil well drilling fluid, PAAF has excellent temperature and complex saline resistance. Under complex saline-based mud, the FL_API_ is 13.2 mL/30 min after aging at 150 °C for 16 h, reaching the industry standard requirement (≤25 mL/30 min).

Compared with hydrolyzed polyacrylonitrile sodium salt (Na-PAN), which is also a small molecular copolymer filtrate reducer, PAAF has a better resistance in complex salts containing calcium and magnesium ions. Compared with another small molecular copolymer filtrate reducer, hydrolyzed polyacrylonitrile ammonium salt (NH_4_-PAN), PAAF has a better filtration-loss-controlling performance. Compared with polycarboxylic acid comb copolymer PAMAP, PAAF has better resistance to saturated brine. Furthermore, PAAF prepared based on low-cost FAS has an advantage in cost and can be used as a low-cost filtrate loss reducer in oil well drilling fluids.

## 4. Materials and Methods

### 4.1. Materials

Fumaric acid sludge (FAS) was obtained from Karamay Zhengcheng Co., Ltd. (Xinjiang, Karamay, China). 2-acrylamido-2-methylpropanesulfonic acid (AMPS) was purchased from Jingwen Dongxin Biotechnology Co., Ltd. (Beijing, China). Acrylamide (AM) was purchased from Shandong Duofeng Chemical Co., Ltd. (Shandong, Zibo, China). potassium persulfate, sodium hydroxide, sodium carbonate, sodium bicarbonate, sodium chloride, anhydrous calcium chloride, magnesium chloride, fumaric acid, maleic acid, etc., were purchased from Beijing Yili Fine Chemicals Co., Ltd. (Beijing, China).

### 4.2. Methods

#### 4.2.1. Preparation of PAAF Based on FAS

FAS pretreatment: FAS was dried in an oven at 80 °C for 24 h, and then crushed into a fine powder with a pulverizer, that is, the fine powder of FAS.

According to the best ratio, 15.0 g NaOH and 5 g Na_2_CO_3_ were added to 80.0 g deionized water and dissolved fully, then 20.0 g FAS fine powder was added slowly and dissolved fully before use; 20.0 g AM and 20.0 g AMPS were added to 40.0 g deionized water and dissolved fully before use; weigh 0.6 g of K_2_S_2_O_8_ initiator and 3.0 g of Na_2_SiO_3_ cross-linking agent into 10.0 g of deionized water, dissolve fully before use. The FAS solution was mixed with the AM solution and the AMPS solution, and then transferred to a three-necked flask to obtain a mixed solution. The temperature of the mix solution was raised to 55 °C while N_2_ was passed into the flask for 30 min. Then, slowly add the K_2_S_2_O_8_ initiator mixture dropwise to the three-necked flask to initiate the polymerization. After 5 h of reaction, a brown viscous liquid was obtained, which was completely dried in an oven at 80 °C. Finally, the dried liquid was pulverized into a fine powder with a pulverizer to obtain PAAF.

#### 4.2.2. Performance Evaluation Method of PAAF

**Figure 11:**
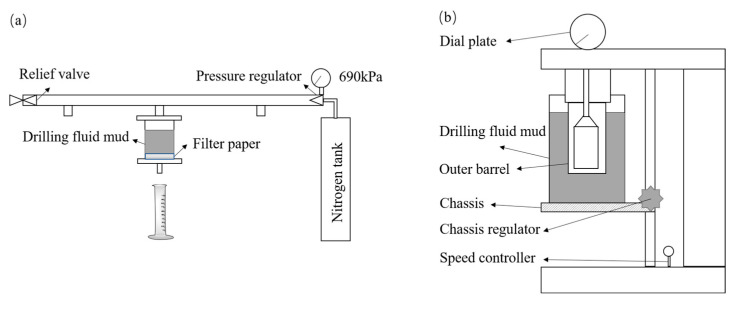
Test schematic: (**a**) FL_API_; (**b**) rheological properties.

The method of GB/T 16783.1 “Field Testing of Drilling Fluids for Oil and Gas Industry Part 1: Water-based Drilling Fluids” was adopted to measure the room temperature filtration loss (FL_API_), high-temperature and high-pressure filtration loss (FL_HTHP_) and rheological properties of PAAF [38]. In addition, the temperature and complex saline resistance of PAAF was tested according to the technical requirements of the corporate standard “Salt Resistant filtrate loss reducer FRS for Drilling Fluids”, issued by China National Offshore Oil Corporation (CNOOC) [36]. The specific test methods are as follows: 

Method of room temperature filtration loss FL_API_: Pour the drilling fluid sample into the filtration loss meter cup, make the liquid level reach the scale line in the filtration loss meter cup, put the filtrate paper and install the filtration loss meter, and put the dry measuring cylinder underneath to receive the filtrate. Close the pressure relief valve and adjust the pressure regulator to make the pressure reach 690 ± 35 kPa (100 ± 5 psi) in 30 s or less, and start timing while pressurizing. After reaching 30 min, measure the collected filtrate volume, which is the room temperature filtration loss FL_API_, and the test schematic is shown in Figure 11a.

Method of the rheological properties: Pour the drilling fluid sample into the sample cup of the rotational viscometer, make the liquid level reach the scale line in the sample cup of the rotational viscometer, put the sample cup on the bottom frame of the viscometer, move the bottom frame so that the sample liquid level coincides with the scale line on the outer cylinder, and measure and record the temperature of the drilling fluid sample. Adjust the rotational speed of the outer cylinder of the rotational viscometer, and after the dial reading value is stabilized, read and record the dial reading value under different rotational speeds, as shown in Figure 11b. Calculate the apparent viscosity, plastic viscosity and dynamic shear force based on the following equation:

AV = R_600_/2 (1)

PV = R_600 −_ R_300_ (2)

YP = 0.511(R_300 −_ PV) (3)

RYP = YP/PV(4)

n = 3.322l g(R_600_/R_300_) (5)

K = (0.511 R_300_)/511^n^ (6)

where:

R_600_ = the reading of the viscometer at 600 r/min (dia);

R_300_ = the reading of the viscometer at 300 r/min (dia);

AV = apparent viscosity (mPa·s);

PV = plastic viscosity (mPa·s);

YP = yield point (Pa);

RYP = the ratio of yield point to plastic viscosity (Pa/mPa·s);

n = fluidity index of power law model rheological equation (dimensionless quantity);

K = the consistency coefficient of power law model rheological equation (Pa·s^n^).

Method of temperature and complex saline resistance evaluation: Complex saline was prepared by adding 45.0 g of sodium chloride, 5.0 g of anhydrous calcium chloride and 13.0 g of magnesium chloride per liter of distilled water. Add 1.0 g of sodium bicarbonate and 35.0 g of evaluation clay to 350 mL of complex saline and stir at high speed for 20 min, stopping at least twice during the period to scrape off the clay adhering to the walls of the container (maintenance at 25 ± 3 °C 24 h). Two portions of the prepared base mud were added with a certain mass of PAAF and stirred at high speed for 20 min. One portion, after being maintained for 24 h, was taken out and stirred at high speed for 10 min, and then the room temperature filtration loss FL_API_ was determined, along with FL_HTHP_ and rheological parameters. The other portion was loaded into a high-temperature tank and hot rolled at 150 °C for 16 h; after it had cooled, it was taken out and stirred at high speed for 10 min, and then the room temperature filtration loss FL_API_ was determined, along with FL_HTHP_ and rheological parameters.

In this test process, a hot roll furnace XGRL-5 was used for hot rolling and aging the drilling fluid, a medium-pressure ZNS-2A was used to determine FL_API_, and high-pressure filtration apparatus GGS42-2 was used to determine FL_HTHP_. The rheological parameters, such as AV, PV, and YP, were determined by a ZNN-D6 rotating viscometer. All of the equipment was made by Qingdao Haitongda Special Instruments Co., Ltd., Qingdao, China.

#### 4.2.3. Fourier Transform Infrared Spectra (FT-IR)

Sample preparation of PAAF was achieved using the KBr pellet method, and the sample was scanned by infrared spectrometer (Nicolet IS5; PerkinElmer; Waltham, MA, USA) in a wave number range of 400 to 4000 cm^−1^, a signal-to-noise ratio of 50,000:1. The infrared spectrum of PAAF samples was recorded at a resolution of 4 cm^−1^ with 64 scans.

#### 4.2.4. Thermogravimetric Analysis (TGA)

The thermal decomposition behavior of the PAAF samples was investigated using thermogravimetric analysis (STA449F5; NETZSCH; Selb, Bavaria, Germany) under nitrogen(N_2_) atmosphere, and the N_2_ flow rate was 40 mL/min. The PAAF samples were placed in a clean crucible and heated from 25 to 350 °C at a ramp rate of 10 °C/min.

## Figures and Tables

**Table 1:** Component composition of FAS.

Component Name	Mass Content/%	CAS#	Carboxylic Acid Saturation	Carboxylic Acid Type
Fumaric acid	19.82	110-17-8	Unsaturation (33.69%)	Diprotic (51.04%)
Fumaric anhydride	0.07	108-30-5		
Maleic acid	13.11	110-16-7		
Maleic anhydride	0.62	108-31-6		
Acrylic acid	0.07	79-10-7		
Phthalic acid	17.10	88-99-3	Saturation (64.21%)	
Phthalic anhydride	0.25	85-44-9		
Benzoic acid	3.71	65-85-0		Mono
Acetic acid	0.15	64-19-7		
Citric acid	43.02	77-92-9		Ternary
Silicate	0.25	-	-	-
Water	1.85	-	-	-

**Table 2:** Effect of cross-linking agent on the performance of PAAF.

Mass of Sodium Silicate (g)	AV (mPa·s)		PV (mPa·s)		YP (Pa)		FL_API_ (mL/30 min)	
	Before Aging	After Aging	Before Aging	After Aging	Before Aging	After Aging	Before Aging	After Aging
0.0	10.0	4.0	10.0	4.0	0.0	0.0	3.6	22.0
1.5	8.5	3.0	8.0	3.0	0.5	0.0	6.8	16.0
3.0	7.0	3.5	6.0	3.0	1.0	0.5	6.4	13.2
6.0	7.0	3.0	6.0	3.0	1.0	0.0	9.6	66.0
12.0	7.0	3.5	6.0	3.0	1.0	0.5	12.4	74.0

**Table 3:** Rheological property results of the complex saline-based mud with the addition of PAAF110 or PAAF111.

Temperature/ °C	Index	Complex Saline-Based Mud	
		With PAAF110	With PAAF111
25	AV/ mPa·s	20.5	8.0
	PV/ mPa·s	19.0	7.0
	RYP/(Pa/mPa·s)	0.0807	0.1486
	n/ Dimensionless	0.8981	0.8301
	K/ Pa·s^n^	0.0415	0.0260
150	AV/ mPa·s	7.0	3.5
	PV/ mPa·s	6.0	3.0
	RYP/(Pa/mPa·s)	0.1703	0.1703
	n/ Dimensionless	0.8074	0.8074
	K/ Pa·s^n^	0.0266	0.0133

**Table 4:** FL comparison of different types of filtrate loss reducers in different based mud (after aging at 150 °C).

Filtrate Loss Reducer	Saturated Brine-Based Mud		Complex Saline-Based Mud	
	FL_API_/mL	FL_HTHP_/mL	FL_API_/mL	FL_HTHP_/mL
Standard [36]	≤25.0	-	≤25.0	-
PAAF	14.2	62.4	13.2	126.0
Na-PAN	7.2	25.6	94.0	250.0
NH_4_-PAN	32.0	250.0	110.0	250.0
PAMAP	15.8	49.4	-	-

## Data Availability

Data are available from the authors upon request.
